# Cobalamin deficiency in pregnancy

**DOI:** 10.1002/ccr3.4282

**Published:** 2021-06-23

**Authors:** Heleen Konings, Yves Jacquemyn

**Affiliations:** ^1^ Global Health Institute Department of Obstetrics and Gynaecology Antwerp University Hospital UZA Antwerp University UA Antwerp Belgium

**Keywords:** homocysteine, methylmalonic acid, pregnancy, scobalamin

## Abstract

Hydroxycobolamine supplementation in hereditary cobolamine deficiency and serial biochemical follow‐up allow uncomplicated pregnancy outcome.

## Graphical abstract

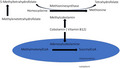

## 1 INTRODUCTION

Cobalamin deficiency is a rare disorder in the remethylation pathway of homocysteine, resulting in homocysteine and methylmalonic acid accumulation. We report a pregnancy in a woman with known cobalamin C/D deficiency treated with hydroxocobalamin.

**FIGURE 1:**
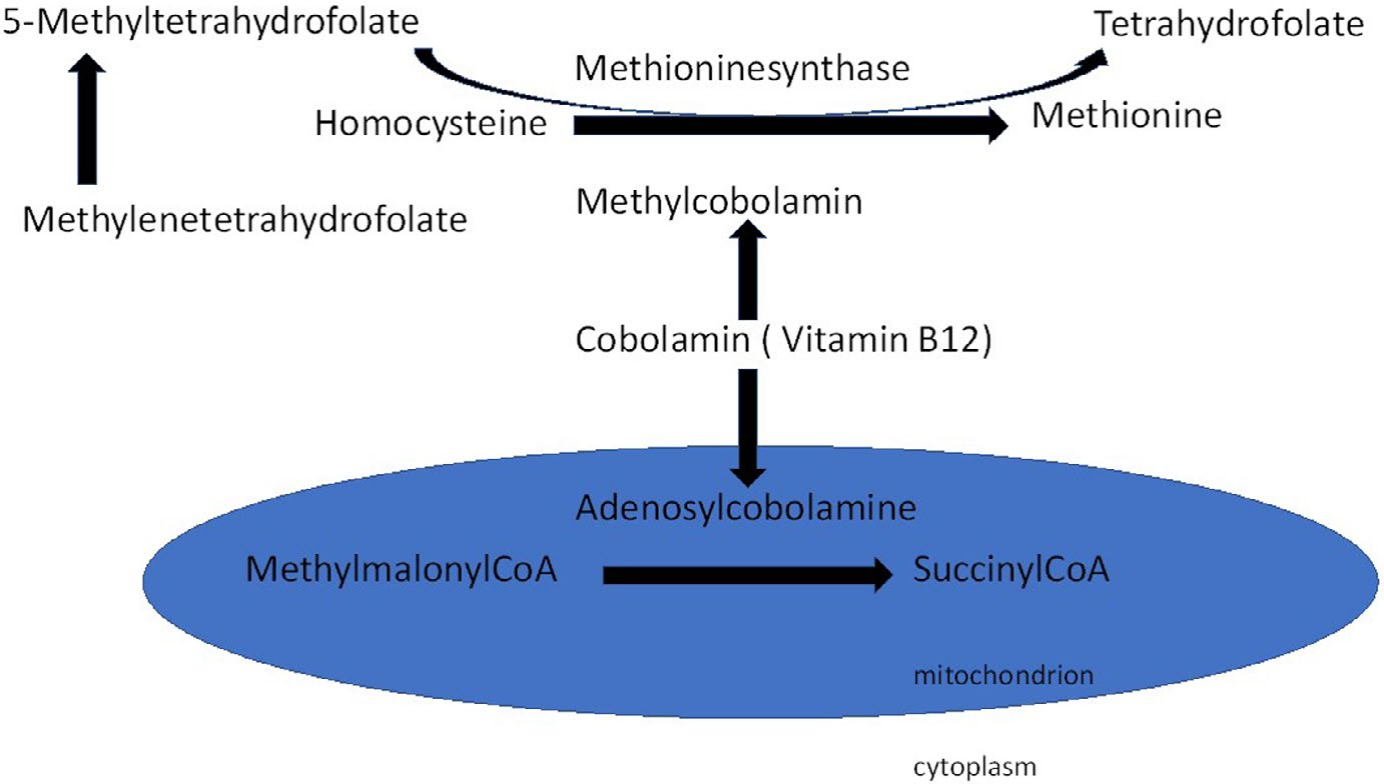
Schematic overview of biochemical pathways in which cobalamin is involved

Cobalamin (vitamin B12) defects are rare, autosomal recessive inherited disorders in the general population causing defects in the remethylation pathway of homocysteine. Homocysteine is an amino acid derived from methionine. Under normal circumstances, homocysteine is subsequently converted into cysteine or it is remethylated, forming methionine again. Inherited cobalamin deficiency is a remethylation defect causing the accumulation of homocysteine and methylmalonic acid (Figure 1).^1^, ^2^ Cobalamin C deficiency (cblC type; OMIM#277400) is the most common inborn error of cobalamin metabolism and involves mutations in the MMACHC gene. The disease shows considerable genetic and clinical heterogeneity, with over 40 different mutations identified. Most cases present during childhood (early onset), but symptoms can also develop in adulthood (late onset). Symptoms include visual impairment due to retinopathy, neurocognitive development delay, pulmonary arterial hypertension, thrombotic microangiopathy and skin depigmentation. Adult onset is associated with mild symptoms involving muscle weakness, thrombosis, intestinal and bladder function problems and neurologic dysfunction, including psychosis and dementia. Bioechemically, patients demonstrate low plasma cobalamin (<150 pg/mL), methylmalonic aciduria and hyperhomocysteinemia; all can be normalized by cobalamin supplementation.

There are very few cases reported of cobalamin deficiency in pregnancy. We report a case of pregnancy in a patient with cobalamin C/D deficiency treated with hydroxocobalamin.

## 2 CASE HISTORY

A primigravid 31‐year‐old woman visited our clinic at 16 weeks gestation. She was diagnosed with a cobalamin C/D deficiency at the age of 28 through family screening after her 26 year old sister was diagnosed with clbC while suffering from debilitating muscle weakness. Our patient had no symptoms, and there were no neurologic abnormalities found on clinical examination; despite high plamsa homocysteine (80 microml/L, normal <15 micromol/L) and high urinaru methylmalonic acis (364 mmol/mL, normal: 0 mmol/mL) at the first visit. No other family members reported any symptoms, there were no other siblings in the family.

Because of the recessive inheritance pattern of this disorder, her partner underwent genetic testing and he turned out not to be a carrier of cobalamin deficiency. This implicates that the baby has a minimal risk of having the same deficiency.

For her cobalamin deficiency, she was treated with intramuscular injections of hydroxocobalamin 100 mg every 2 weeks, which she continued throughout the entire pregnancy. During pregnancy, she smoked two cigarettes a day. Ultrasound investigations at 16, 23, and 35 weeks gestation showed no structural abnormalities. Fetal growth was normal for the given gestational age. Metabolic follow‐up at 19 weeks gestation demonstrated undetectable homocysteine values in blood and urine.

Right before delivery, a blood test given showed a hemoglobin value of 11 g/dL, a slightly elevated value of white blood cells (18 400/µL), normal ionogram value, and decreased creatinine value (0.40 mg/dL), all within the physiologic ranges for pregnancy. The value of vitamin B12 was >2000 ng/L, and methylmalonic acid was undetectable in urine. This implicated a control of the cobalamin C/D deficiency. The patient has always refused to follow any dietary restrictions.

At 37 weeks and 6 days gestation, the patient went into spontaneous labor leading to a vaginal delivery. There were no complications, and on the same day, she delivered a term daughter of 2.230 kg (<10th percentile on the Flemish growth charts) with a length of 46 cm (50th percentile) and a head circumference of 33 cm (50th percentile). APGAR scores after 1, 5, and 10 minutes were 9, 10, and 10, respectively. A neonatal physical examination showed no abnormalities.

Postpartum, the patient was observed for 5 days to ensure there were no problems concerning her cobalamin deficiency. During this period, there were no specific problems reported, and she was able to go home in good condition. Ammonia levels in the blood were elevated (27 µmol/L) the day after delivery, and they decreased to 21 µmol/L on the second day. After delivery, carnitine (carnitine syrup 1000 mg per day) was added to the treatment. At 3 months postpartum, her metabolic follow‐up was normal.

## 3 DISCUSSION

Different remethylation disorders are inherited in an autosomal recessive way, all of which result in deficient activity of methionine synthase (Figure 1). These disorders are characterized by increased serum levels of homocysteine and by decreased levels of methionine through different defects. There can be a decrease in the function of methionine synthase itself.^1^, ^2^ This is called cobalamin E or G (cblE or G) deficiency, and it is the result of defective methyl‐cobalamin (MeCbl) synthesis.^2^ There can also be a decreased supply of the substrate methylenetetrahydrofolate (MTHF), which results in impaired 5‐methyltetrahydrofolate (CH3‐THF) synthesis. This is called 5, 10‐methylenetetrahydrofolate reductase (MTHFR) deficiency.^1^, ^2^ Defects in intracellular cobalamin (cbl) transport and processing can also be the causes of a remethylation deficiency. These are called cblC, D, F, and J deficiencies, with cblC being the most common.^1^, ^3^ They disturb not only the diversion of vitamin B12 (or cobalamin) into MeCbl but also its diversion into adenosylcobalamin (AdoCbl).^1^, ^4^ MeCbl and AdoCbl are important cofactors for methylmalonyl‐CoA mutase and methionine synthase.^4^ These last‐described genetic disorders are thus characterized by accumulations of homocysteine and methylmalonic acid (MMA).^1^, ^2^ Regarding vitamin levels in the blood, measured serum vitamin B12 is usually normal or high.^2^

Diagnosing a remethylation disorder in patients using the clinical picture might be difficult due to the highly heterogeneous presentation.^1^, ^3^ These conditions can affect multiple systems, resulting in neurological, hematological, and ophthalmological abnormalities, including seizures, movement disorders, neuropathy, pigmentary retinopathy, and macrocytic anemia. In some patients, renal pathology (glomerulopathy or atypical hemolytic uremic syndrome [HUS]) is also reported.^1^

In 1998, the European Journal of Pediatrics published guidelines for diagnosing and treating remethylation disorders. They stated that patients with a new cobalamin deficiency should first be treated with intravenous hydroxocobalamin and, after a few weeks, with intramuscular injections (1‐2 mg/dose). They also state that the addition of betaine (3‐6 g/day), folic acid (5 mg/day), and carnitine (50‐100 mg/kg/day) can be beneficial.^2^ In 2016, the Journal of Inherited Metabolic Disease published guidelines for diagnosing and managing cobalamin‐related methylation disorders. When preparing their recommendations, they used the GRADE approach for a quality assessment of the evidence (low–moderate–high). When comparing these guidelines, the Journal of Inherited Metabolic Disease also strongly recommended parenteral hydroxocobalamin treatment with no delay following suspicion of a remethylation disorder (moderate). Treatment with oral betaine is also recommended (low). The use of folic acid or carnitine, however, is controversial. No beneficial effects have been reported, nor have deleterious consequences (low) been reported. They also recommend not starting a restricted protein diet (moderate).^1^

The main treatment of cobalamin deficiency is hydroxocobalamin, which is used instead of cyanocobalamin because it seems more effective in patients with cblC deficiency.^1^ The mechanism behind this response is not yet understood. The gene responsible for cblC deficiency is the MMACHC gene. Froese et al^5^ describe that MMACHC with the R161Q mutation binds with more affinity to hydroxocobalamin than to cyanocobalamin. Betaine supplementation is recommended because of its involvement in the remethylation process, resulting in a decrease in homocysteine and an increase in methionine. Betaine catabolism, however, is dependent on folate. Thus, folic acid supplementation could be considered in long‐term betaine therapy, but its effectiveness is not proven.^1^, ^2^ Carnitine levels may be low in cblC patients, because the de novo synthesis of carnitine relies on methionine. Therefore, supplementation of carnitine can be considered, but its benefit is not proven.^1^, ^2^

Our patient was treated with hydroxocobalamin and carnitine. The treatment with hydroxocobalamin was already initiated before conception, as she was receiving intramuscular injections of hydroxocobalamin 100 mg (1000 µg) of every 2 weeks. After delivery, she also received carnitine 1000 mg/day. This is a general measure we follow to counter the high metabolic demand. Her treatment did not include betaine.

The main concern regarding metabolic disorders in pregnancy lies in the increase in amino acids. When they exceed the catalytic capacity of the enzymes needed for their metabolism, there is an accumulation of toxic metabolites, which could be harmful for the mother and the baby. Especially, the involution of the uterus postpartum leads to an increase in proteins and, thus, the risk of an accumulation of metabolites, such as ammonia.^6^

Few studies have been published concerning cobalamin deficiency in pregnancy; as far as we were able to determine, this case report is the fifth one published. Three previous case reports on pregnant women with cobalamin C (cblC) deficiency reported healthy neonatal outcomes and no maternal complications during pregnancy and postpartum. In two cases, the women started having treatment at 15 weeks gestation. One of the women was treated with a low protein diet, acetylsalicylic acid (80 mg/day), cobalamin, folic acid, and carnitine.^7^ The other woman had treatment in the form of cobalamin, folic acid, l‐carnitine, and betaine.^8^ In the third case, the cblC defect was only discovered after delivery. Screening of the carnitine levels in the newborn were low and gave rise to the identification of a cblC defect in the asymptomatic mother after further research. The baby itself was unaffected.^9^ There is one report published of a pregnant woman with cobalamin A deficiency who was treated with hydroxocobalamin before and during pregnancy. Her pregnancy was uneventful and the baby was born healthy.^6^

In most cases, complementation studies to identify the genetic defect of the cobalamin deficiency are not accomplished. Therefore, these patients are categorized as having a cblC/D deficiency.^4^ This explains why the patient we described in this case is not labeled as having either a cblC or a cblD deficiency.

Guidelines for the management of cobalamin deficiency in pregnancy do not exist. We emphasize serial monitoring and clinical and biochemical observation as the mainstays of management. Careful observation, anticipation, and management of maternal and fetal complications are key considerations.

## CONFLICT OF INTEREST

None declared.

## AUTHOR CONTRIBUTIONS

HK: involved in case description and reviewed the literature. YJ: involved in discussion and performed the data collection on the case.

## CONSENT STATMENT

Published with written consent of the patient.

## Data Availability

The authors confirm that the data supporting the findings of this study are available within the article, no funding has been provided for this article and local ethical guidelines have been followed.
